# Non-professional phagocytosis: a general feature of normal tissue cells

**DOI:** 10.1038/s41598-019-48370-3

**Published:** 2019-08-15

**Authors:** Jacob C. Seeberg, Monika Loibl, Fabian Moser, Manuela Schwegler, Maike Büttner-Herold, Christoph Daniel, Felix B. Engel, Arndt Hartmann, Ursula Schlötzer-Schrehardt, Margarete Goppelt-Struebe, Vera Schellerer, Elisabeth Naschberger, Ingo Ganzleben, Lucie Heinzerling, Rainer Fietkau, Luitpold V. Distel

**Affiliations:** 1 Department of Radiation Oncology, University Clinic Erlangen, Friedrich-Alexander-Universität Erlangen-Nürnberg, Erlangen, 91054 Germany; 2 Experimental Renal and Cardiovascular Research, Department of Nephropathology, Friedrich-Alexander-Universität Erlangen-Nürnberg, Erlangen, 91054 Germany; 3 Department of Pathology, University Clinic Erlangen, Friedrich-Alexander-Universität Erlangen-Nürnberg, Erlangen, 91054 Germany; 4 University Clinic Erlangen, Friedrich-Alexander-Universität Erlangen-Nürnberg, Erlangen, 91054 Germany; 5 Department of Medicine 4 – Nephrology and Hypertension, University Clinic Erlangen, Friedrich-Alexander-Universität Erlangen-Nürnberg, Erlangen, 91054 Germany; 6 Department of Surgery, University Clinic Erlangen, Friedrich-Alexander-Universität Erlangen-Nürnberg, Erlangen, 91054 Germany; 7 Department of Medicine 1, University Clinic Erlangen, Friedrich-Alexander-Universität Erlangen-Nürnberg, Erlangen, 91054 Germany; 8 Department of Dermatology, University Clinic Erlangen, Friedrich-Alexander-Universität Erlangen-Nürnberg, Erlangen, 91054 Germany

**Keywords:** Entosis, Immune cell death

## Abstract

Non-professional phagocytosis by cancer cells has been described for decades. Recently, non-professional phagocytosis by normal tissue cells has been reported, which prompted us to take a closer look at this phenomenon. Non-professional phagocytosis was studied by staining cultured cells with live-cell staining dyes or by staining paraffin-embedded tissues by immunohistochemistry. Here, we report that each of 21 normal tissue cell lines from seven different organs was capable of phagocytosis, including *ex vivo* cell cultures examined before the 3rd passage as well as the primary and virus-transformed cell lines. We extended our analysis to an *in vivo* setting, and we found the occurrence of non-professional phagocytosis in healthy skin biopsies immediately after resection. Using dystrophin immunohistochemistry for membrane staining, human post-infarction myocardial tissue was assessed. We found prominent signs of non-professional phagocytosis at the transition zone of healthy and infarcted myocardia. Taken together, our findings suggest that non-professional phagocytosis is a general feature of normal tissue cells.

## Introduction

The engulfment of cells by neighbouring, non-professional phagocytic cells has received growing attention in recent years. It is commonly believed that living cells engulf other living or dying cells. In cancer cells, high rates of cell-in-cell structures are observed, called cannibalism^1^. Most frequently, cancer cells internalise other cancer cells or heterotypic inflammatory cells, which is called emperipolesis^2^. In contrast, entosis is the active invasion of a live cell into another cell^3^. To date, cancer cells have been studied regarding their ability to engulf other cancer cells. It is not clear whether apoptotic or necrotic cells are preferentially engulfed or whether both types of dead cells are phagocytosed with similar efficiency. We recently showed that necrotic or necroptotic cancer cells are engulfed by homotypic or heterotypic live cells^4^. Additionally, we demonstrated that primary skin fibroblasts internalise necrotic fibroblasts^5^. The phagocytosis of apoptotic or necrotic cells by phagocytes is called efferocytosis^6^. Mostly, efferocytosis is linked to professional phagocytes such as macrophages^7–10^, while additional non-professional phagocytic cells may act as phagocytes. Reports in the literature exist about cells from normal tissue cell lines engulfing other normal tissue cells. Normal tissue cells denote healthy, non-neoplastic tissue cells. Additionally,

non-professional phagocytosis was observed in normal tissues by smooth muscle cells, renal cells and hepatocytes^11–14^. Consequently, the question arises as to whether phagocytosis is a common feature of all normal tissue cells. We studied the occurrence of non-professional phagocytosis in 21 different normal tissue cell lines, including skin tissue and myocardial infarction specimens.

## Results

### *In vitro* homotypic phagocytosis

Our aim was to investigate the ability of normal tissue cells to phagocytise other necrotic normal tissue cells. We studied 21 normal tissue cell lines from seven different organs. The cells were stained with green or red fluorescent dyes. The cells stained red were heated to 56 °C for 30 minutes to induce necrosis (Supplementary Data, Fig. S1). These necrotic cells were then co-incubated with viable cells from the same cell line and were compared to viable cells that were co-incubated with viable cells. The fraction of phagocytised cells was analysed by fluorescence microscopy. The occurrence and frequency of cell-in-cell structures (CICs) was defined as CICs per 1000 living cells.

**Figure 1:**
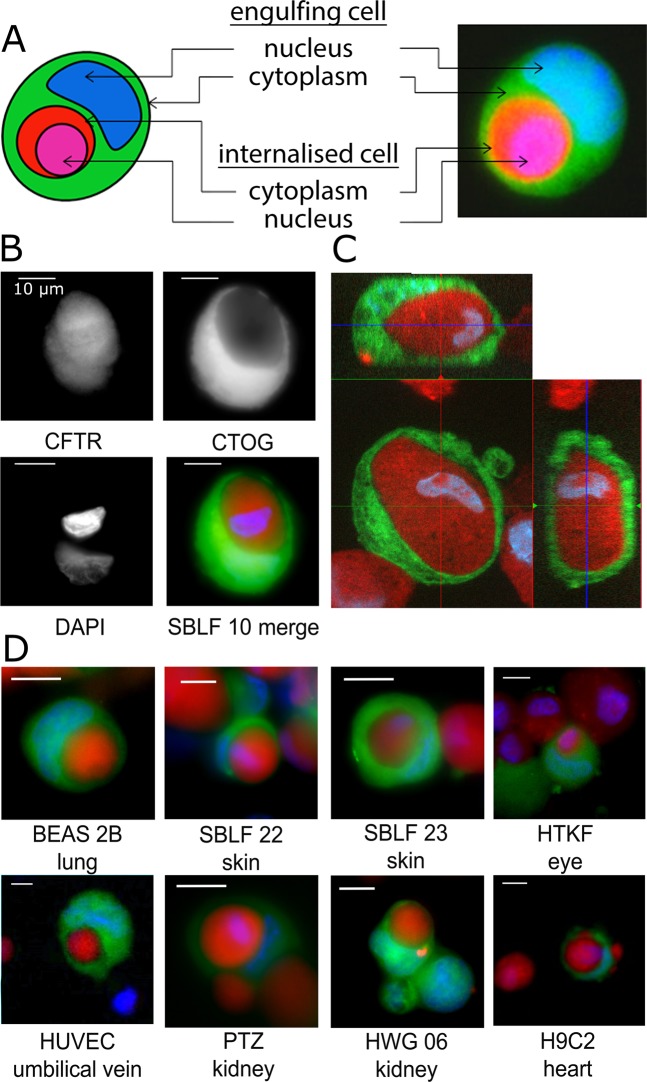
A schematic view and representative CIC images. The engulfing green cell contains an internalised, round, red cell and a crescent-shaped compressed nucleus (**A**). A CIC structure assembled by the internalised CFTR (red)-labelled cell and the engulfing CTOG (green)-labelled cell. The nucleus is labelled with DAPI and appears blue (**B**). A z-stack image of an SBLF 10 fibroblast cell (green) engulfing a dead cell (red). The nuclei are stained blue. The left panel is a xy optical slice taken at approximately the midpoint of the cell. The top left is an xz cross-section, and the right is a yz cross-section (**C**). Eight representative CIC images derived from different cell lines are displayed (**D**). Scale bars in are 10 µm.

A CIC was counted when a red cell with a round shape and an intact nucleus was fully engulfed by a green cell that displayed a nucleus compressed into a crescent shape by the internalised red cell (Fig. 1A–C, Supplementary Data, Figs S2 and S3).

**Figure 2:**
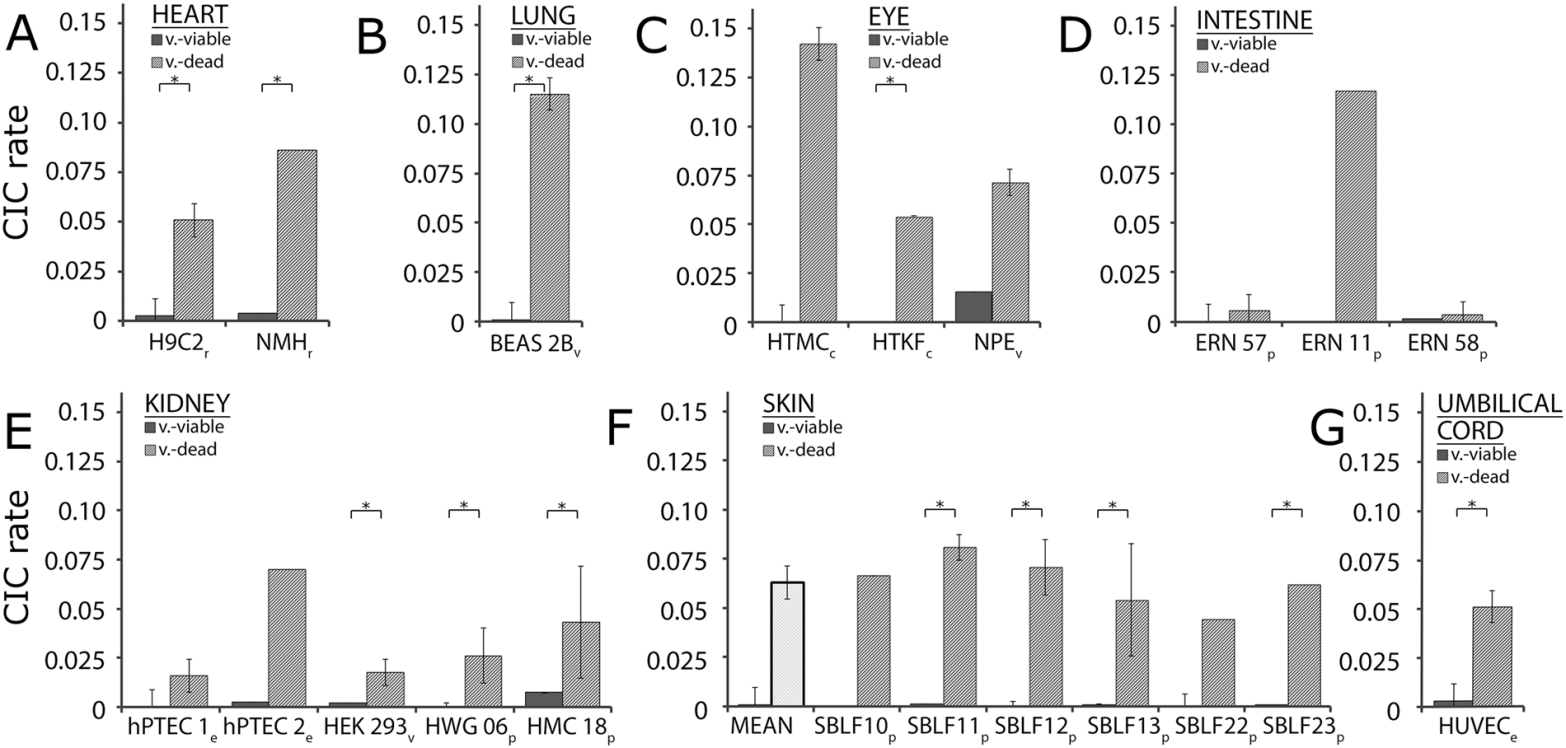
The cell-in-cell rates in 21 different cell lines of different tissues, comparing the viable cells incubated with dead cells or with other viable cells. The CICs in cell lines obtained from the heart (**A**), lung (**B**), eye (**C**), intestine (**D**), kidney (**E**), skin (**F)** and umbilical cord (**G**). p indicates cell lines of primary origin that were cultivated for less than ten passages; e indicates *ex vivo* cell lines used prior to the fifth passage; v marks commercially available, virus-transformed cell lines; c indicates commercially available cell lines. *Indicates p < 0.05.

We found CICs in every studied cell line at varying frequencies (Fig. 2). CIC frequencies were constantly higher in the viable-dead (intervention) group compared to that in the viable-viable (control) group (p < 0.05). Non-myocytic cells from rat hearts (Rattus Norvegicus) formed CICs in up to 8.6% (Fig. 2A), meaning that approximately 1/10 of all viable cells engulfed a necrotic cell. A comparable rate of CICs was observed in the immortalised human lung epithelial cell line BEAS-2B with

11.5% necrotic cells internalised (Fig. 2B). The two connective tissue and one non-pigmented epithelium-derived eye cell lines phagocytised between 5.4% and 14.2% of cells, respectively (Fig. 2C). Primary large intestinal fibroblast cell cultures engulfed 11.7% of cells in a single experiment (Fig. 2D). All kidney-derived cells formed distinct CICs. The *ex vivo* hPTEC cells formed up to 7% CICs in a single experiment. The frequency of CICs in the two primary human mesangial and one transfected embryonic cell lines ranged from 1.7% to 4.3% (Fig. 2E). We studied six primary cell lines from skin biopsies that phagocytised necrotic cells at a frequency of 4.4 to 8.0%. CICs occurred in a frequency of 6.3% among the skin fibroblasts of 6 individuals of variable age and gender (Fig. 2F). A total of 5.1% CICs were observed in human umbilical vein endothelial cells in the viable-dead (intervention) group and 0.3% in the viable-viable (control) group (Fig. 2G).

### CIC in skin biopsies

**Figure 3:**
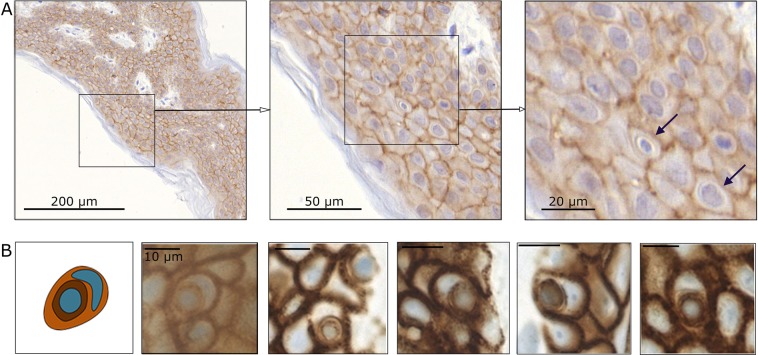
Non-professional phagocytes in the skin. Skin biopsies were immunohistologically stained for the cell membrane protein E-cadherin (brown) and for cell nuclei (blue) (**A**). A schematic view and examples of cell-in-cell structures in skin tissue are shown (**B**). Scale bars in **(A**) are 200 µm, 50 µm, and 20 µm; scale bars in (**B**) are 10 µm.

In previous experiments we demonstrated that all studied normal tissue cell lines have the ability to phagocytise necrotic cells. Next, we used *ex vivo* skin discs to study the occurrence of CICs in healthy, complex tissue. We found structures fulfilling all criteria of a fully engulfed cells (CIC) in all skin biopsies (Fig. 3A,B). We assessed 190,000 cells from five samples and found a mean CIC frequency of 0.128 +/− 0.128 CIC per 1000 cells. The highest CIC rate found was 0.38 CIC per 1000 cells.

### CIC in myocardial infarction

**Figure 4:**
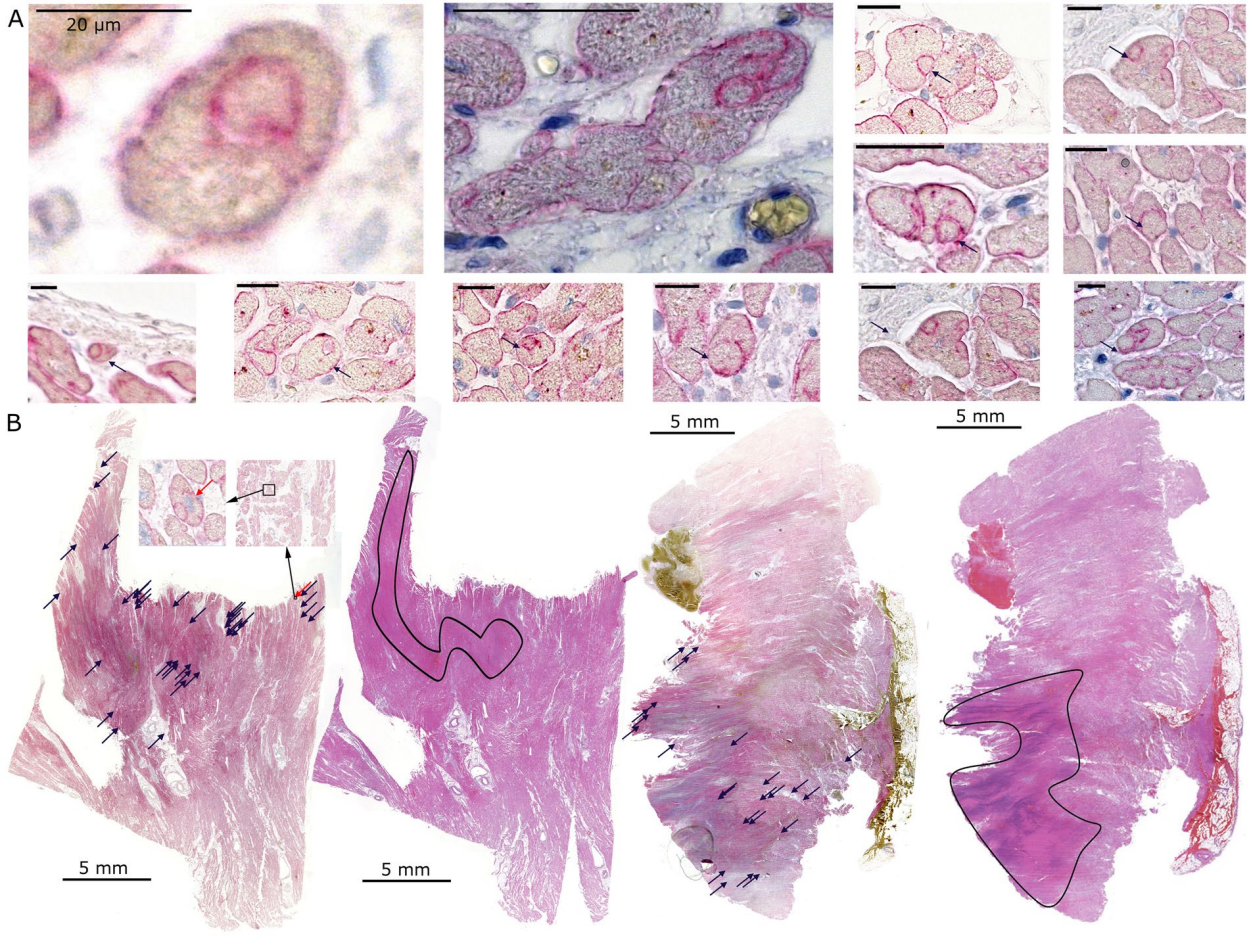
Non-professional phagocytes in heart tissue. Examples of CIC structures in the myocardium stained by dystrophin and haemalaun (**A**). A human post-infarction myocardium slide was stained for the cell membrane protein dystrophin and the neighbouring slide was stained with haematoxylin and eosin (**B**). The CIC-like structures were marked on the dystrophin slide (blue arrows). Afterwards, the infarction area was detected in the haematoxylin and eosin-stained slides (black outline). Inset images show two magnifications of the indicated area. The red arrows mark an exemplary CIC-like structure. The scale bars in (**A**) are 20 µm and in (**B**) are 5 mm.

We systematically searched for CICs in dystrophin-stained slides before locating the infarction areas. Several membrane inclusions with CIC-like features were present without meeting exactly all of our criteria of a CIC (Fig. 4A, Supplementary Data, Fig. S4). We found internalised cells displaying round nuclei surrounded by their cell membrane, which were completely encircled by the host cells. However, the crescent-shaped nuclei of the phagocytic cells were mostly not visible. After matching the infarction areas with the CIC-like structures, we found these structures to be located predominantly in the transition zone between the infarction and healthy myocardia with a frequency of 0.144 +/− 0.082 CIC per mm^2^ (Fig. 4B). In the control tissue of healthy myocardium, we found these membrane structures to be spread throughout the tissue without any recognisable pattern at a frequency of 0.031 +/− 0.041 CIC per mm^2^.

## Discussion

Our finding that cells from all 21 cell lines from the non-neoplastic tissue of seven different organs perform phagocytosis indicates that a broad number of normal tissue cells from different origins have the capability to phagocytose other necrotic cells. We use the term “normal tissue” to refer to healthy and non-neoplastic tissue cells or tissues. Additionally, in skin tissue, cells were phagocytosed, and CIC-like structures were distributed around the infarction zone of patients with myocardial infarction. We postulate that non-professional phagocytosis is a mechanism that could take place in nearly all cells of every tissue. The question arises as to why normal tissue cells should have the ability to phagocytose necrotic neighbouring cells. The reason may be that necrotic dying cells promote inflammation and harm their neighbouring cells by releasing damage-associated molecular patterns, cellular contents or otherwise sequestered intracellular moieties^15,16^. Non-professional phagocytosis may participate in the limitation of these mechanisms, beginning before a sufficient number of macrophages have immigrated to the injured region^17^. In the absence of macrophages, non-professional phagocytes are responsible for the clearance of apoptotic cells^18,19^.

Among the 21 phagocytic normal tissue cell lines, we studied virus-transformed cell lines, long established cell lines, primary cell lines and freshly isolated kidney cells. All cell lines had the ability to phagocytose, which hints at a general phagocytic ability of cells. However, it cannot be ignored that the special experimental setting favors phagocytosis. Therefore, we established our *ex vivo* skin tissue model. The aim was to substantiate the occurrence of CICs in normal tissue in which no injury caused extensive cell death. Our finding of CICs in these samples underlined the significance of non-professional phagocytosis in healthy skin. We assume cells were engulfed due to necrotic or apoptotic cell death, though other reasons are possible. We cannot rule out that the surgical trauma or additional factors may have promoted cell damage. Therefore, compared with that in *in vivo* conditions, the CIC rate in these conditions might be elevated, yet the occurrence of CIC was clearly shown. Following these results and speculations, we tried to study the occurrence of CICs in a situation requiring high phagocytic activity. Tissue infarction can lead to the simultaneous formation of countless necrotic and apoptotic events; thus, myocardial infarction was chosen as a representative setting. Our research on myocardial tissue showed multiple CIC-like structures. Although these structures failed to fulfil all CIC criteria, the cell membrane configurations and round shapes of the cells represent core CIC features.

The missing crescent-shaped nuclei of the phagocytic cells may be lacking because of the relatively large cardiomyocytes and the low probability of slicing through the nucleus of the host cells. These CIC-like structures are spread evenly among the healthy myocardial samples but group along the transition zone between the infarction and the healthy tissue in post-infarction myocardial samples. CIC-like structures have also previously been described in infarcted rat hearts, even though the authors were not aware of the phenomenon of CICs^20^. Recently, it was shown that cardiac myofibroblasts play an important role in the clearance of necrotic and apoptotic cells after myocardial infarction. It was postulated that these phagocytic cells complement professional phagocytes^17^. Our findings suggest the involvement of myocytes in the phagocytosis and clearance of damaged cells after a myocardial infarction. Similarly, it was demonstrated that non-professional phagocytosis by the bronchial epithelium plays a critical role in airway inflammation^18^. Quite recently, the importance of non-professional phagocytosis by hepatocytes in various liver diseases and in healthy tissue has been confirmed^14^.

In contrast to normal tissue, the formation of CIC structures is well known in cancer. This is often called cell cannibalism and is found in a broad range of malignant tumours, such as medulloblastoma^21^, breast carcinoma^22^, lung cancer^23^, head and neck cancer^4,24^, gastric carcinoma (Caruso, Muda *et al*. 2002), pancreatic adenocarcinoma^25^, rectal and anal carcinoma^4^ and bladder cancer^26^. It is apparent that cancer cells possess phagocytosis abilities. The reason why CICs are regularly found in cancer may be the high frequency of necrotic or apoptotic cell death. Additionally, cancer tissue is regularly assessed by pathologists, and therefore CICs are noticed. In normal tissue, the rate of dying cells is much less frequent, resulting in a much lower frequency of CICs. Furthermore, normal tissue is rarely examined; thus, cell-in-cell structures are less likely to be detected. This may be the reason why this general feature of nearly all cell types is not known as well.

Taken together, our findings, in accordance with the recent literature, indicate that non-professional phagocytosis is a common mechanism in normal tissue cells to remove dying cells, complement professional phagocytes and prevent the release of proinflammatory cell contents.

## Methods

### Cell culture

Nineteen human and two rat cell lines were studied *in vitro* for their homotypic phagocytosis capability. The human cell lines were derived from various tissue sites and donors of mixed ages and genders. Two *ex vivo* cell lines (from two renal tissues), 16 primary cell lines and three virus-transformed cell lines were studied. We used epithelial cells, fibroblasts, endothelial cells, mesangial cells and muscle cells in our experiments (Supplementary Table 1). The cells were cultured in individually composed media containing varying amounts of F12-Medium (Life Technologies GmbH, Darmstadt, Germany), Dulbecco’s Modified Eagle’s Medium (DMEM, Pan-biotech, Life Technologies GmbH, Darmstadt, Germany), foetal bovine serum (FBS-Cambrex, Verviers, Belgium), further supplements and 1% penicillin/streptomycin antibiotics medium (Life Technologies GmbH, Darmstadt, Germany). Formulations are displayed in Supplementary Table 1.

### Cell lines from seven different normal tissues

Five different kidney cell lines were studied. The tubular renal cell lines hPTEC 1 and 2 were studied directly after isolation from the donors’ organs within three passages. The cells were isolated by manual dissection, collagenase digestion and cell straining^27^. They were provided by the Department of Medicine 4. Adenovirus 5-immortalised human embryonic kidney cells^28^ and two primary cell lines were derived from whole glomeruli (HWG06) and human mesangial cells (HMC18) (<20th passage), respectively, and were provided by the Department of Nephropathology, Erlangen. Human umbilical vein endothelial cultures were isolated from umbilical cords. The cells were cultured with increased levels of CO_2_ (7.5% vs. the standard 5%). Only the first passage was used. The heart cells originated from rats (Rattus Norvegicus). Non-myocytic heart cells were a mixture of cardiac endothelial cells and fibroblasts^29^. Embryonic cardiomyocytes (H9C2; American Type Culture Collection ATCC CRL 1446) were isolated from the cardiac ventricle. Both cell lines were provided by the Experimental Renal and Cardiovascular Research workgroup at the Department of Nephropathology, University Clinic Erlangen. Three human eye cell lines were studied. Human tenon capsule fibroblasts (HTKFs) were isolated from normal donor eyes. The dissection and collagenase digestion were followed by centrifugation and cultivation. Human trabecular meshwork cells (HTMC) were isolated from the juxtacanalicular and corneoscleral region of the human trabecular meshwork tissue. They are commercially available at Provito, Berlin (6590).

In addition, an SV40 virus-transformed^30,31^ non-pigmented ciliary epithelial (NPE) cell line from a 27-year-old male donor was utilised^32,33^. All ocular cells were obtained from the Department of Ophthalmology, University Clinic Erlangen. We studied three primary normal fibroblast cell cultures from the human large intestine. ERN 11, ERN 57 and ERN 58 were all isolated from healthy tissue, at least 10 cm away from colorectal cancer tissue in resected colon segments. The tissue was minced, enzymatically digested (collagenase 2; Biochrom AG, Berlin), meshed and harvested by centrifugation. The fibroblasts were cultured after magnetic cell sorting (MACS; Miltenyi Biotech, Bergisch Gladbach).

The cells were grown in an 8.5% CO_2_ humid atmosphere at 37 °C. We studied six consecutive fibroblast primary cell cultures isolated from healthy human Caucasian donors. The isolation was performed by harvesting the emigrating fibroblasts from flask-cultured skin biopsies. BEAS-2B, human bronchial epithelium cells were obtained from the United Kingdom Sigma/Public Health Consortium. This epithelial-like growing cell line has been transformed by the Ad12 (ad12) and simian virus 40 (SV40) hybrid virus. The cell line was established in 1988^34^. The cell lines are not listed in the International Cell Line Authentication Committee (ICLAC) database.

### Cell staining and immunofluorescence

The phagocytosis assay was performed using the following dyes: CellTrace Far Red, CellTracker Red, CellTracker Oregon green (Invitrogen, Auckland, New Zealand) and DAPI (Roche, Grenzach-Whylen, Germany). The cell samples were permeabilised for staining with DAPI and were fixed at room temperature using 3.9% formaldehyde/0.5% Triton X-100 (Merck, Darmstadt, Germany) in phosphate-buffered saline (Life Technologies, Darmstadt, Germany). The cells were washed three times in Tris-buffered saline (Carl Roth, Darmstadt, Germany). All slides were mounted using Vectashield + DAPI mounting medium (Vector Laboratories Inc., Burlingame, USA). The experiments using skin biopsies were treated as follows: the tissue was stained by an E-cadherin antibody (mouse IgG2a, BD Bioscience, Heidelberg, Germany). The antigen retrieval was performed in EDTA buffer, pH 8.0 (1.5% 2-butoxyethanol, 5% sodium EDTA) in a pressure cooker (PC6–25; Nesco, Two Rivers, WI, USA) for 1 minute at 120 °C. The antigen signal amplification was performed by a streptavidin-biotinylated alkaline phosphatase complex (Vectastain ABC HRP Kit, Vector Laboratories Inc, Burlingame, USA). The cell nuclei were stained with haemalaun.

### Non-professional phagocytosis assay *in vitro*

The phagocytic activity in the cell lines was studied as follows: A cell population was split into four parts, two of which were stained red, and two were stained green. Necrosis was induced in one red (viable-dead, intervention group) population in a hyperthermic (56 °C) bath for 30 minutes. Routinely, we performed 7AAD flow cytometry to ensure sufficient induction of necrosis (Supplementary Data Fig. S1). Equal numbers of red and green cells were co-incubated for 5 h. The remaining red and green populations (viable-viable, control group) were co-incubated simultaneously and served as controls. After fixation and permeabilisation, slide areas of 2 mm^2^ were scanned with a semi-automatic microscope (Axiovert 200, Carl Zeiss Microscopy, Göttingen, Germany) at 400× magnification. The whole images were analysed with Biomas image-processing software (MSAB, Erlangen, Germany). Cell-in-cell (CIC) phenomena were counted as fractions of all viable green cells. A given structure had to fulfil the following criteria to be marked as a CIC: The internalised (red) cell displayed an intact nucleus and was round due to lack of cell junctions, and the engulfing cell (green) displayed a crescent-shaped nucleus compressed by the phagocytised cell (Fig. 1A–C). All experiments were in accordance with protocols approved by the Institutional Review Committee of the University Erlangen-Nürnberg. All methods were carried out in accordance with the relevant guidelines and regulations. The statistical analysis was performed with SPSS 19. For statistical testing, the Mann-Whitney U test was used.

### Human samples

Experiments with human samples were performed according to the regulations of the ethical boards of the University Clinics of Erlangen Nuremberg, and written informed consent was obtained from all individuals. The use of skin disks (204 17 Be) and the isolation of human renal cells (Reference number 3755) was approved by the Ethics Review Committee of the University Erlangen-Nürnberg and was approved by the local ethics committee. The use of formalin fixed paraffin-embedded material from the Archive of the Institute of Pathology was approved by the Ethics Committee of the Friedrich-Alexander-University of Erlangen-Nuremberg on 24 January 2005, waiving the need for consent for using the existing archived material.

### CIC in skin biopsies

The occurrence of CICs in skin biopsies of healthy individuals was studied. Five skin biopsies of approximately 10 cm^2^ each were obtained from the Department of Dermatology of the University Clinic Erlangen. Excess skin was used, which was leftover during surgery for other reasons. The patients were of mixed age and gender, and the skin was removed from different locations. After extraction, the skin biopsy was fixed immediately in formaldehyde. All samples were paraffin-embedded and were processed into tissue slides; the cell membranes were stained for E-cadherin (anti-CD324, BD Biosciences, Heidelberg, Germany). CICs were detected manually by the use of imaging software.

### CIC in myocardial infarction

We investigated the occurrence of CICs in myocardial post-infarction tissue. Myocardial tissue from the post-mortem analysis of twelve patients who died of myocardial infarction was obtained and processed for immunohistochemistry. The infarction age was between 12 and 48 h. Tissues from 5 patients with non-cardiovascular causes of death were assessed as a control. We identified the infarction area, transition zone and healthy myocardium by haematoxylin and eosin staining. In a neighbouring slide, we used an anti-dystrophin antibody (MAB 1645, Merck KGaA, Darmstadt, Germany) to stain the myocardial cell membranes. We used a Mirax MIDI Slide Scanner (Carl Zeiss MicroImaging GmbH, Göttingen, Germany) to capture images of the immunohistochemistry slides. A CIC search was conducted manually. A total of 3960 mm^2^ (22 slides) of myocardial post-infarction tissue and 1696 mm^2^ (11 slides) of myocardial tissue from patients with non-cardiovascular deaths were analysed.

## Supplementary information

Supplementary Information
