# Author Correction: Non-professional phagocytosis: a general feature of normal tissue cells

**DOI:** 10.1038/s41598-020-65963-5

**Published:** 2020-06-04

**Authors:** Jacob C. Seeberg, Monika Loibl, Fabian Moser, Manuela Schwegler, Maike Büttner-Herold, Christoph Daniel, Felix B. Engel, Arndt Hartmann, Ursula Schlötzer-Schrehardt, Margarete Goppelt-Struebe, Vera Schellerer, Elisabeth Naschberger, Ingo Ganzleben, Lucie Heinzerling, Rainer Fietkau, Luitpold V. Distel

**Affiliations:** 1 Department of Radiation Oncology, University Clinic Erlangen, Friedrich-Alexander-Universität Erlangen-Nürnberg, Erlangen, 91054 Germany; 2 Experimental Renal and Cardiovascular Research, Department of Nephropathology, Friedrich-Alexander-Universität Erlangen-Nürnberg, Erlangen, 91054 Germany; 3 Department of Pathology, University Clinic Erlangen, Friedrich-Alexander-Universität Erlangen-Nürnberg, Erlangen, 91054 Germany; 4 University Clinic Erlangen, Friedrich-Alexander-Universität Erlangen-Nürnberg, Erlangen, 91054 Germany; 5 Department of Medicine 4 – Nephrology and Hypertension, University Clinic Erlangen, Friedrich-Alexander-Universität Erlangen-Nürnberg, Erlangen, 91054 Germany; 6 Department of Surgery, University Clinic Erlangen, Friedrich-Alexander-Universität Erlangen-Nürnberg, Erlangen, 91054 Germany; 7 Department of Medicine 1, University Clinic Erlangen, Friedrich-Alexander-Universität Erlangen-Nürnberg, Erlangen, 91054 Germany; 8 Department of Dermatology, University Clinic Erlangen, Friedrich-Alexander-Universität Erlangen-Nürnberg, Erlangen, 91054 Germany

Correction to: *Scientific Reports* 10.1038/s41598-019-48370-3, published online 15 August 2019

This Article contains an error. In Figure 4 A, one of the representative images is included twice. A correct version of Figure 4 appears below as Figure 1.

**Figure 1:**
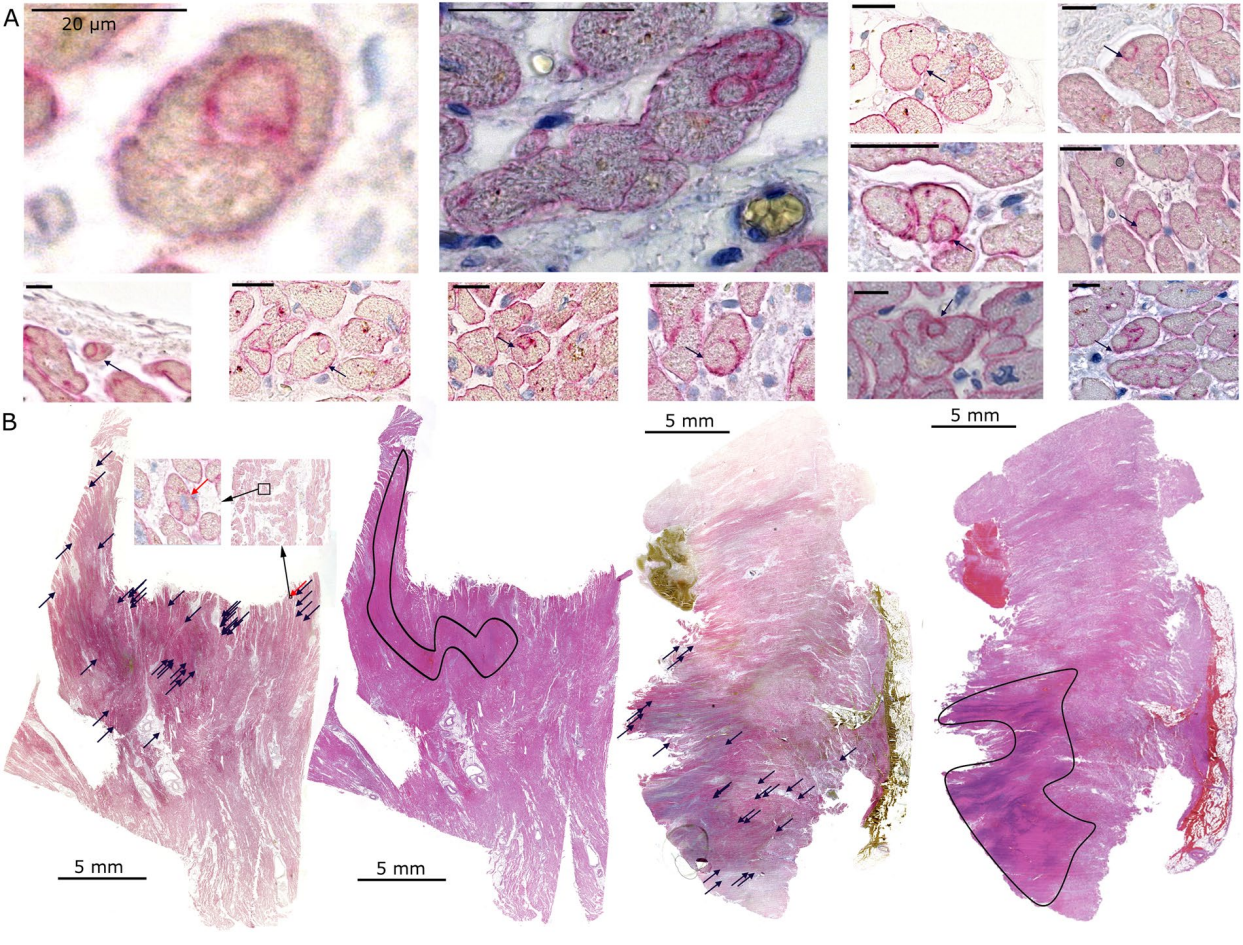
.

