# Supplementary material for: Non-professional phagocytosis: a general feature of normal tissue cells
Source: Sci Rep. 2019 Aug 15;9:11875. doi: 10.1038/s41598-019-48370-3 (PMC6695441; doi:10.1038/s41598-019-48370-3)
Supplement: Supplementary file 1 — Supplementary Information [file 41598_2019_48370_MOESM1_ESM.pdf]

Non-professional phagocytosis: a general feature of normal tissue cells

Jacob C. Seeberg[1], Monika Loibl[1], Fabian Moser[1], Manuela Schwegler[1], Maike Büttner-Herold[2], Christoph Daniel[2], Felix B. Engel[2], Arndt Hartmann[3], Ursula Schlötzer-Schrehardt[4], Margarete Goppelt-Struebe[5], Vera Schellerer[6], Elisabeth Naschberger[6], Ingo Ganzleben[7], Lucie Heinzerling[8], Rainer Fietkau[1], and Luitpold V. Distel[1,*]

[1]Department of Radiation Oncology, University Clinic Erlangen, Friedrich-Alexander-Universität Erlangen-Nürnberg, Erlangen, 91054, Germany
[2]Experimental Renal and Cardiovascular Research, Department of Nephropathology, Friedrich-Alexander-Universität Erlangen-Nürnberg, Erlangen, 91054, Germany
[3]Department of Pathology, University Clinic Erlangen, Friedrich-Alexander-Universität Erlangen-Nürnberg, Erlangen, 91054, Germany
[4]University Clinic Erlangen, Friedrich-Alexander-Universität Erlangen-Nürnberg, Erlangen, 91054, Germany
[5]Department of Medicine 4 – Nephrology and Hypertension, University Clinic Erlangen, Friedrich-Alexander-Universität Erlangen-Nürnberg, Erlangen, 91054, Germany
[6]Department of Surgery, University Clinic Erlangen, Friedrich-Alexander-Universität Erlangen-Nürnberg, Erlangen, 91054, Germany
[7]Department of Medicine 1, University Clinic Erlangen, Friedrich-Alexander-Universität Erlangen-Nürnberg, Erlangen, 91054, Germany
[8]Department of Dermatology, University Clinic Erlangen, Friedrich-Alexander-Universität Erlangen-Nürnberg, Erlangen, 91054, Germany
[*]Luitpold.distel@uk-erlangen.de

Supplementary Table

Supplementary Table 1: Cells and medium

Manufacturers of the cells and media: DMEM, PAN-Biotech; GmbH, Aidenbach, Germany; high glucose DMEM GlutaMAX, L-Glutamine, HAMS F12 and penicillin/streptomycin, Life Technologies GmbH, Darmstadt, Germany; foetal bovine serum and non-essential amino acids, Biochchrom AG, Berlin, Germany; endothelial cell growth medium and supplement mix, PromoCell GmbH, Heidelberg, Germany.

Name of cell cultures/lines	Type	Organ	Cell type	Medium	Fetal bovine serum	Additive(s)	Source
BEAS 2B	Virus- transformed	Lung	Epithelial	DMEM GlutaMAX	10%	1% Penicillin/Streptomycin	Department of Medicine 1
ERN 57, 11, 58	Primary cultures	Large intestine	Fibroblasts	DMEM GlutaMAX	10%	1% Penicillin/Streptomycin	Department of Surgery
HEK 293	Virus- transformed	Kidney	Embryonic	DMEM	10%	1% Penicillin/Streptomycin	Department of Nephropathology
HMC 18	Primary cultures	Kidney	Mesangial	DMEM	10%	1% Penicillin/Streptomycin	Department of Nephropathology
HTKF	Primary cultures	Eye	Fibroblasts	DMEM/HAMS F12	15%	1% Penicillin/Streptomycin	Department of Ophthalmology
HTMC	Primary cultures	Eye	Trabecular	DMEM	10%	1% Penicillin/Streptomycin	Department of Ophthalmology
HUVEC	Ex vivo cultures	Umbilical vein	Endothelial	Endothelial Cell Growth Medium	-	2% Supplement mix	Department of Nephropathology
HWG 06	Primary cultures	Kidney	Mesangial	DMEM	10%	1% Penicillin/Streptomycin	Department of Nephropathology
H9C2	Primary cells (rat)	Heart	Myocyte	DMEM GlutaMAX	10%	1% Penicillin/Streptomycin	Department of Nephropathology
NMH	Primary cells (rat)	Heart	Fibroblasts/ Endothelial	DMEM GlutaMAX	10%	1% Penicillin/Streptomycin	Department of Nephropathology
NPE	Virus- transformed	Eye	Epithelial	DMEM GlutaMAX	10%	1% Penicillin/Streptomycin	Department of Ophthalmology
hPTEC 1, 2	Ex vivo cell cultures	Kidney	Proximal tubule epithelial	DMEM/HAMS F12	2.5%	1% Penicillin/Strep.<br>1% L-Glutamine<br>1% Inulin Transferrin<br>17μl Hydrocortison<br>200μl Triiodthyronin<br>50μl Epidermal growth factor	Department of Medicine 4

SBLF 10-13, 22, 23	Primary cultures	Skin	Fibroblasts	HAMS F12	15%	1% Penicillin/<br>1% L-Glutamine<br>2% Non-Essential Amino Acids	Department of Radiation Oncology

The use of non-commercially available cell lines was approved by the Ethics Committee of the University Erlangen-Nürnberg. Below the Reference numbers of the Ethics Committee of the University Erlangen-Nürnberg are displayed:

- Human renal cells (HWG06, HMC18, hPTEC, HEK293), Reference number 3755, TS-05/11
- Human skin fibroblasts (SBLF 10-13, 22,23) Reference number 2575
- Human eye cells (HTKF) Reference number 4218-CH
- Intestinal cells (ERN 11,57,58) Reference number 3402

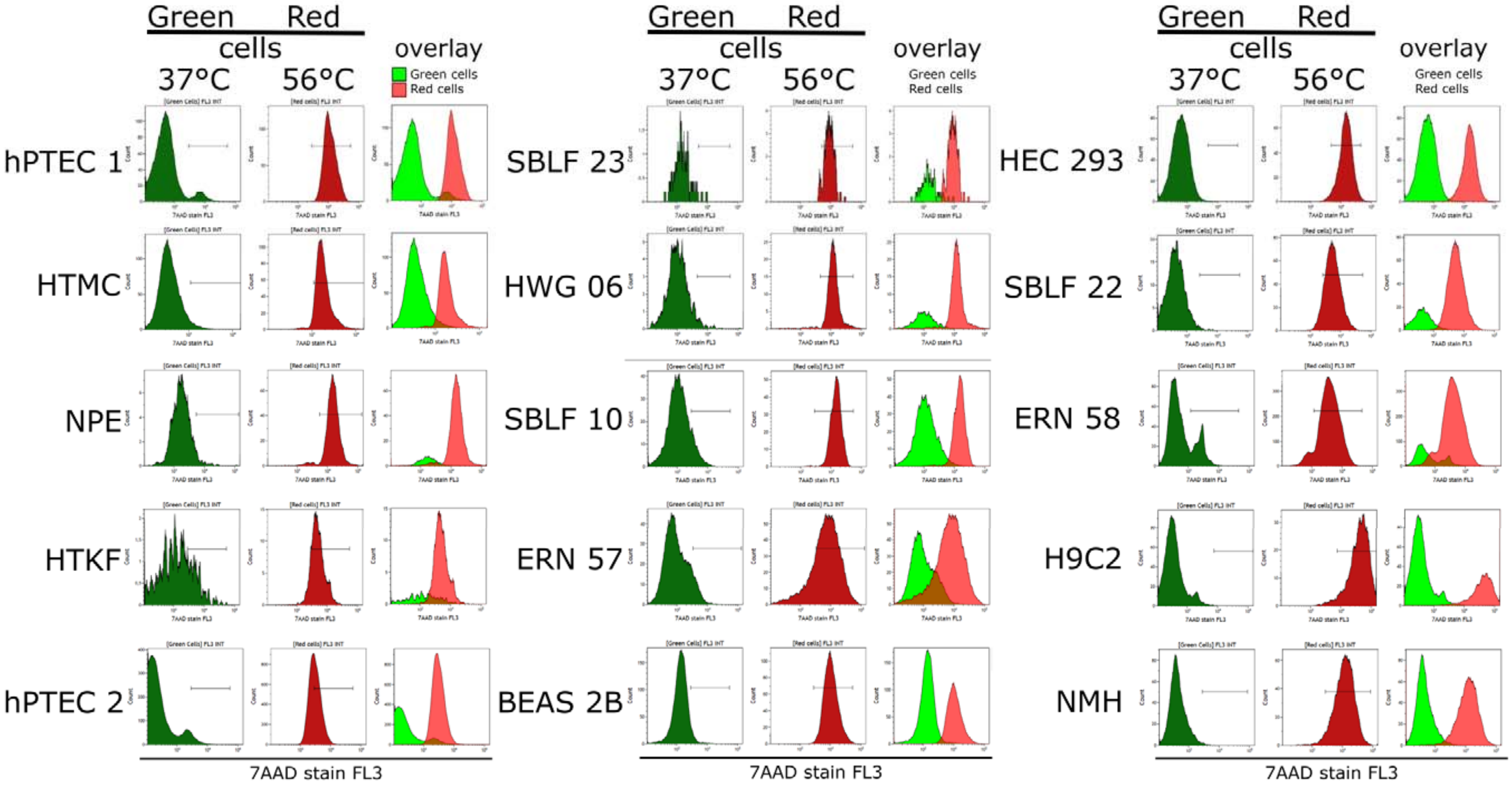

Supplementary Figure S1. Routine 7AAD flow cytometry charts. We assessed 7AAD uptake by necrotic (red) and healthy (green) cell populations. In all cases, more than 65% of the red cells were necrotic, as seen by flow cytometry. Cells were stained for the non-professional phagocytosis assay. One part of the cells was stained by the cell tracing dye CTOG (green) and one part by CTFR (red). Red cells were heated at 56°C for 30 minutes. 7AAD was added on ice for 30 minutes. CTOG was acquired in FL1 channel, 7AAD in FL3 channel and CTFR in FL6 channel.

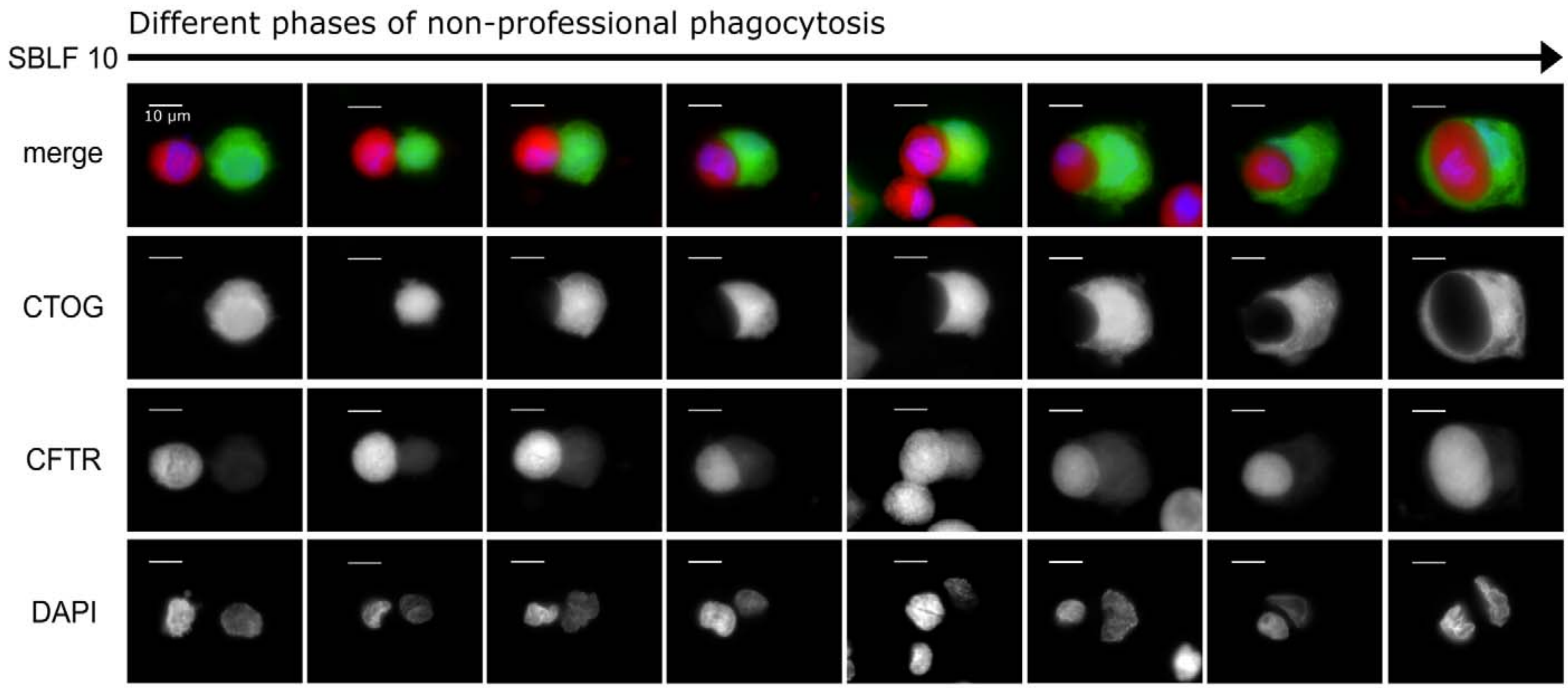

Supplementary Figure S2. The different phases of non-professional phagocytosis. Images of the non-professional phagocytosis process of SBLF 10 fibroblast cultures. The living cells were stained green (CTOG), and the necrotic cells were stained red (CFTR). The nuclei were stained blue by DAPI. Eight representative CIC images derived from the different phases of non-professional phagocytosis are displayed. The scale bars are 10 µm.

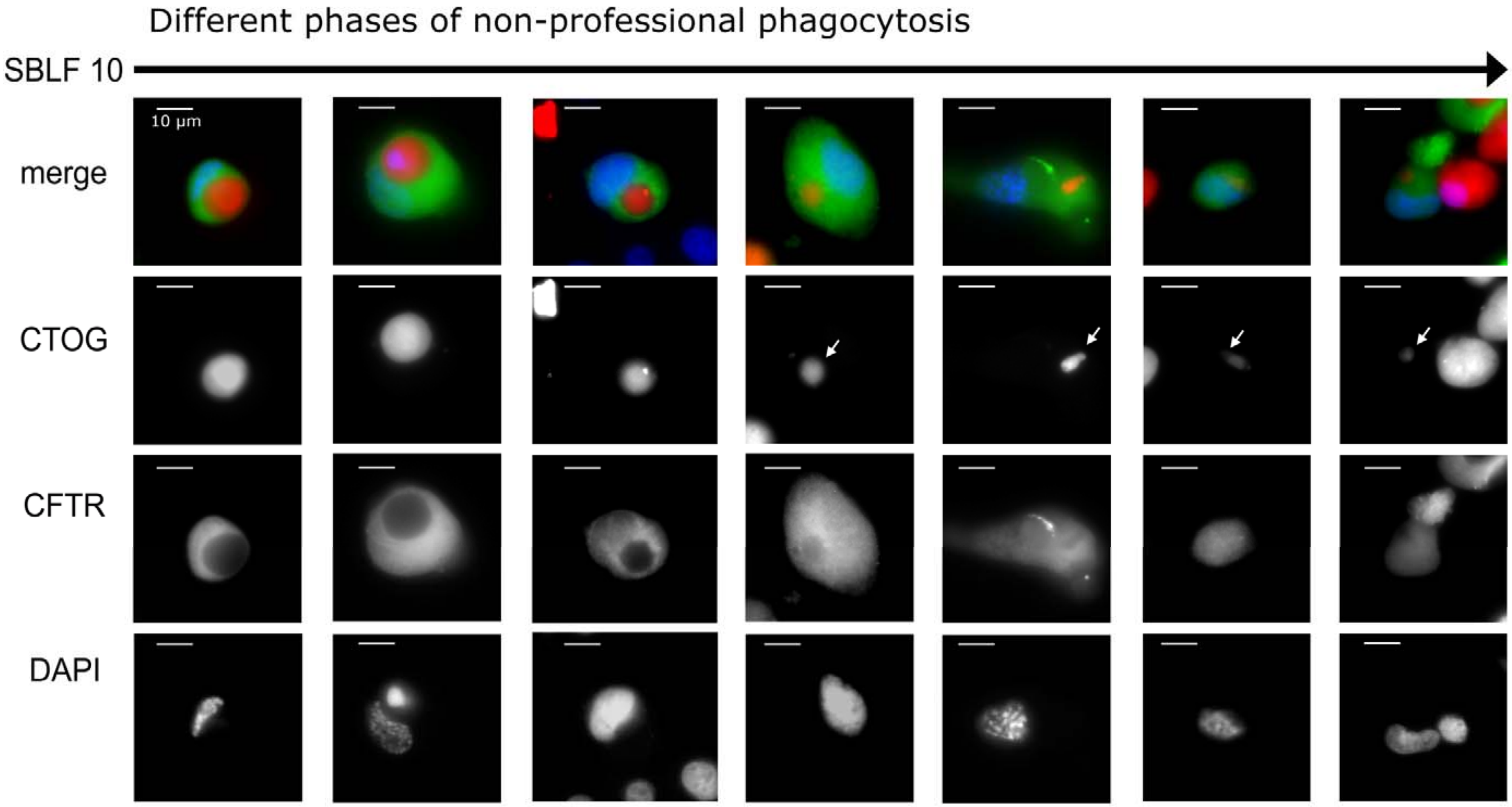

Supplementary Figure S3. The different phases of non-professional phagocytosis. Images of the non-professional phagocytosis process of SBLF 10 fibroblast cultures. The dead cells (red) are indicated by a white arrow. The living cells were stained green (CTOG), and the necrotic cells were stained red (CFTR). The nuclei were stained blue by DAPI. Seven representative CIC images derived from the different phases of non-professional phagocytosis are displayed. The scale bars are 10 μm.

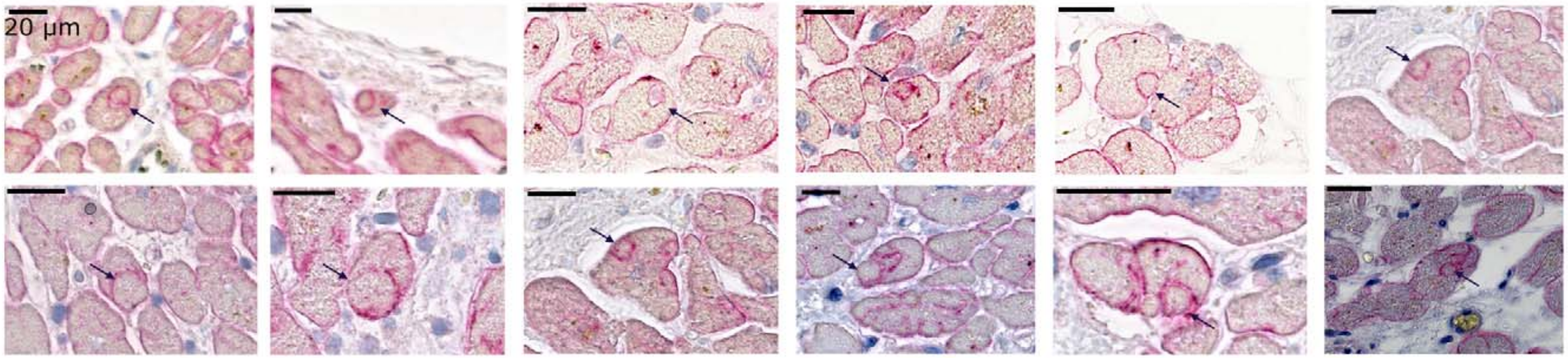

Supplementary Figure S4. Non-professional phagocytes in heart tissue. The examples of CIC-like structures in the myocardium are stained by dystrophin and haemalaun. The CIC-like structures were marked on the dystrophin slide (arrows). The scale bars are 20 µm.
